# Editorial: Advances in understanding and managing dentine hypersensitivity and tooth wear

**DOI:** 10.3389/froh.2025.1766533

**Published:** 2026-01-12

**Authors:** Reisha Rafeek, Alex Milosevic, Andrew Eder

**Affiliations:** 1 School of Dentistry, The University of the West Indies St. Augustine, St. Augustine, Trinidad and Tobago; 2 School of Medicine and Dentistry, University of Lancashire, Preston, United Kingdom; 3 Eastman Dental Institute, University College London, London, United Kingdom

**Keywords:** abrasion, cracked tooth syndrome (CTS), dentine hypersensitivity, erosion, tooth wear

Dentine hypersensitivity (DH) and pathological (or accelerated) tooth wear are increasingly prevalent oral health issues, presenting challenges in diagnosis and management that necessitate evidence-based approaches. The key objectives of this Research Topic included investigating the etiology, advancing diagnostic tools, developing prevention strategies, evaluating management approaches and promoting interdisciplinary collaboration.

The research topics highlight the importance of accurate diagnosis to rule out conditions like cracked tooth syndrome and suggest advanced treatments like diode lasers can enhance the effectiveness of traditional therapies. Some studies also point to various contributing factors, including demographic variables, environmental exposures like chlorinated water and even the abrasivity of acidic toothpastes, emphasizing the need for localized data and patient education on lifestyle and hygiene choices.

Global DH prevalence estimates are highly variable and this can be due to differences in methodology and diagnostic criteria. Most prevalence studies have been conducted in Europe, USA, China and Southeast Asia while studies across the MEA region are scarce. The paper titled “*Evidence-based recommendations for diagnosing and managing dentine hypersensitivity in clinical practice: insights from the Middle East and Africa*” presents data specific to the MEA region compiled by a panel of 12 dental professionals from 8 countries. DH prevalence is reported as ranging from 16.3% in Nigeria to 35.4% in Saudi Arabia and 49.8% in Oman. This demonstrates how relatively common DH is in the MEA region and in many instances exceed those in European or Asian populations. These differences may be explained by dietary habits, lifestyle factors, oral hygiene practices and access to healthcare services. This article also provides a simplified algorithm that clinicians can follow in order to diagnose and manage DH more effectively (Cekici et al.).

Another region from which there is scarce data is the Caribbean region. The article titled “*The Influence of demographic variables on the prevalence and severity of tooth wear in a Trinidadian population*” investigated the prevalence and severity of tooth wear in the Trinidadian adult patient population. Varying factors were analyzed such as age groups, sexes, and the three dominant ethnicities in the Trinidadian population, namely: Afro-Trinidadians, predominantly from Western Africa, Indo-Trinidadians, predominantly from South-East Asia, and a Mixed population of Afro and Indo-Trinidadians. This study demonstrates increasing severity of tooth wear with age and there were higher prevalence rates for males with moderate and severe tooth wear compared with mild wear. There was also a difference among the ethnic groups with Afro-Trinidadians having higher prevalence rates of no wear and lower rates of mild, moderate, and severe wear compared to Indo-Trinidadians. Persons of mixed ethnicity appeared to have the same protective effect as African ethnicity (Marchan et al.).

The diagnosis of DH needs to be accurate to rule out other conditions such as caries and cracked tooth syndrome. The article titled “*Cracked tooth syndrome: a diagnostic dilemma- a mini review*” provides excellent insight and summarizes the current strategies and standardized protocols in diagnosing a cracked tooth (Raj and Singh).

The other 3 articles featured in this research topic focus on treatment and management of DH. Many therapies have been used for management of DH and the emergence of laser therapy is promising. The article titled “*Comparison of the effectiveness of diode laser, fluoride varnish, and their combination in treatment of dentin hypersensitivity: a systematic review of randomized clinical trials*” compared the effectiveness of sodium fluoride (NaF) varnish, Diode Laser (DL), and their combination in reducing DH. This study suggests that a combination of diode laser therapy and NaF varnish may provide superior efficacy and longer-lasting relief over NaF alone (Mohammadian et al.).

A common problem with competitive swimmers is dental erosion due to low pH of chlorinated swimming pool water. The *in-vitro* study titled “*Evaluating the protective effects of mouthguards with neutralizing agents against chlorinated water-induced enamel erosion*” very interestingly showed that mouthguards significantly reduced enamel microhardness loss compared to no mouthguard use and that those mouthguards lined with arginine-fluoride toothpaste showed the least enamel loss, after a swimming simulation, suggesting its potential protective effect (Kitsahawong et al.).

There is a vast amount of research conducted on the abrasivity of toothpastes on dentin. The International Organization for Standardization (ISO) recommends that deionized water is used to prepare the slurries of toothpastes for testing. The diluent used to test toothpaste abrasivity in standard tests may have an impact on their results and the article titled “*Buffer Solution Reduces Acidic Toothpaste Abrasivity Measured in Standardized Tests*” showed that using deionized water as a diluent for *in vitro* toothpaste assessment may not create a realistic pH environment and subsequently may lead to general over-estimation of abrasivity of toothpastes (Zehnder et al.).

